# Measuring appreciation made EA-SI-the development of a short scale to measure experienced appreciation in social interactions at work

**DOI:** 10.3389/fpsyg.2025.1465512

**Published:** 2025-03-24

**Authors:** Maximilian Stefan Resch, Elena Nagelmann, Henrik Bellhäuser

**Affiliations:** Institute of Psychology, Johannes Gutenberg University Mainz, Mainz, Germany

**Keywords:** appreciation, EA-SI work scale, work engagement, burnout, positive work

## Abstract

Experienced appreciation at work is incongruently defined and measured in the scientific literature. Therefore, this article aims to give an overview of different definitions and measures of experienced appreciation at work to clarify the confusing state of research. Then, the new construct, Experienced Appreciation in Social Interactions (EA-SI) at Work, is introduced to counter the incongruency in defining experienced appreciation at work and to provide a reliable and comparable operationalization of the construct. In a second step, the article aims to develop and validate a short scale to measure EA-SI more time-efficiently. To do so, the instrument is derived from the original EA-SI Work Scale considering confirmatory factor analyses, artificial intelligence, and the evaluation of naïve and expert judges based on a sample of *N* = 391 employees. Subsequently, the EA-SI Work Scale (short) – including *k* = 4 items each for colleagues and supervisors as a source of experienced appreciation – is validated in a second independent sample with *N* = 323 participants. The assumptions of its theoretical framework (the Stress as Offense to Self-theory) and the relations between EA-SI and employee work engagement and burnout were tested to validate the short scale. Additionally, its internal consistency, convergent, and discriminant validity were determined. Social support was added as a control variable to test for EA-SI’s incremental predictive value. The results highlight the unidimensional structure of EA-SI and point toward high reliability and validity of the short scale. Conclusively, the limitations and implications of the findings are discussed.

## 1 Introduction

The word “appreciation” describes the recognition and acknowledgment of a value (Schäfer, 2021). Accordingly, there are different focus points on which appreciation can be oriented. For example, appreciation can be felt for one’s life circumstances, for other people (Adler, 2002), or one’s own body (Avalos et al., 2005). Besides the sensation of feeling appreciation for someone or something, one can also receive appreciation from others and feel valued by them in social interactions (Pfister et al., 2020; Stocker et al., 2010).

As Ashforth and Schinoff (2016) pointed out, the context of work represents one of the most important contexts upon which human identity is built. In line with this, recent findings highlighted the relevance of experienced appreciation for employee well-being, satisfaction, and motivation. Employees who felt valued by their colleagues and direct supervisors reported higher global self-esteem and lower feelings of stress (Resch and Bellhäuser, 2025; Semmer et al., 2019). The more employees felt appreciated by others, the higher their work satisfaction (Pfister et al., 2020) and general life satisfaction (Resch and Bellhäuser, 2025), as well as their work engagement were (Al-Aali and Ahmed, 2022; Muntz and Dormann, 2020). Furthermore, employees who experienced less appreciation at work felt more emotionally exhausted (Maslach and Leiter, 2017; Resch and Bellhäuser, 2025).

Despite its relevance, appreciation at work is incongruently defined and operationalized and, therefore, has limited comparability between different articles. Appreciation is used synonymously with constructs such as respect (Carstensen et al., 2021; Decker and Van Quaquebeke, 2014), (social) recognition (Schneickert et al., 2019), reward (Bregenzer et al., 2022), or gratitude (Fagley, 2016). Other articles discuss appreciation as a part of social support (Semmer et al., 2019; Sundin et al., 2007). When explicitly defined as appreciation, the theoretical frameworks differ from a single-factor structure (Semmer et al., 2019) over a four-dimensional understanding (Döring-Katerkamp and Rohrmeier, 2016) to a five-dimensional construct definition (White, 2016, 2017).

This article strives to fulfill three objectives. First, a descriptive overview of how appreciation can be defined and measured will be given using instruments recently applied in research. Second, the newly developed construct *Experienced Appreciation in Social Interactions* (EA-SI) and the EA-SI Work Scale (Resch and Bellhäuser, 2025) will be introduced as an alternative to counter the previously described incongruency as it combines different understandings of appreciation in one integrative model based on the well-established Stress as Offense to Self-theory (Semmer et al., 2019). Finally, this article aims to develop a short scale of the EA-SI Work Scale to enable researchers and practitioners to measure the construct more time-economically. To do so, the short scale will be derived from the original instrument in Study One and subsequently validated in the independent sample of Study Two. Please note that whenever the acronym “EA-SI” is used, the underlying construct is addressed, while the instruments to operationalize the construct are named in full length. This differentiation should make it easier to separate the construct from its operationalization.

## 2 Theory

### 2.1 Definition and operationalization of experienced appreciation at work—overview

As mentioned above, defining the construct “appreciation” in scientific literature is characterized by incongruency. Resch and Bellhäuser (2025) already pointed toward this issue but omitted to give a more detailed overview regarding the various approaches to understanding appreciation at work. To extend the understanding of the underlying research gap, we will give a guiding overview of how the construct has previously been defined and operationalized in literature. Please note that the following selection is not based on a systematic literature review.

One construct that is used synonymously to address and operationalize appreciation is the construct “respect.” Following van Quaquebeke and Eckloff (2010), respect is defined as “an attitude toward other people […] that in return engenders in the target a feeling of being appreciated” (p. 345). Respect can be measured using the *Respectful Leadership scale*. The 12 items of the scale are used to measure respect as a one-dimensional construct (van Quaquebeke and Eckloff, 2010). Participants can answer on a five-point Likert scale ranging from 1 (*not at all*) to 5 (*very much*). With 𝛼 > 0.9, the scale’s reliability is excellent (Blanz, 2023). Although respect and appreciation are theoretically distinguished by van Quaquebeke and Eckloff (2010) they are measured synonymously and interwoven with each other. The operationalization is limited to supervisors as a source of respect.

Besides respect, “appreciation” is equated with the construct “reward” in scientific literature. In line with this, Bregenzer et al. (2022) subsume appreciation in one of the “six areas of worklife” by Leiter and Maslach (1999). Reward is defined as “collegial or managerial recognition, material compensation, and intrinsic enjoyment of the work” (Bregenzer et al., 2022). Hence, appreciation is not understood as an autonomous construct but associated with reward as a proximal construct to measure it. Following this line of thought, appreciation could be measured using the dimension “reward” within the German version of the *Areas of Worklife Scale* (Brom et al., 2015). The instrument includes a total of six dimensions. Four of the 29 items capture the dimension “reward” on a five-point Likert scale from 1 (*strongly disagree*) to 5 (*strongly agree*). With 𝛼 > 0.8, the internal consistency of the subscale is high (Blanz, 2023).

Following Semmer et al. (2019), a third construct that could be synonymously used to cover appreciation is the construct of “social support.” In line with Sundin et al. (2007) and Bregenzer et al. (2022), appreciation could be understood as a subsumed part of social support, which is defined as the “overall levels of helpful social interaction available on the job from both co-workers and supervisors” (Karasek and Theorell, 1990, p. 69). Following this definition, appreciation could be measured with the social support scale by Frese (1989). Five of the 20 items record the perceived social support from colleagues, and five different items survey social support by supervisors. The four-point rating scale ranges from 1 (*not at all*) to 4 (*completely*). The internal consistency is 𝛼 > 0.8 and, therefore, high (Blanz, 2023).

However, Semmer et al. (2019) and Resch and Bellhäuser (2025) pointed out that the construct of experienced appreciation can be theoretically and statistically distinguished from other constructs, such as social support. Therefore, they argue that appreciation should be treated as an autonomous construct.

In line with this understanding, one approach to defining appreciation is the theory of the “five languages of appreciation” by White (2016) including the five dimensions “words of affirmation,” “quality time,” “acts of support,” “material gifts,” and “physical touch.” Theoretically, these dimensions are believed to be unambiguously distinguishable from each other. With the help of the *Motivating by Appreciation Inventory* (MBA Inventory) the languages of appreciation can be measured. As the fifth language did not appear appropriate within the work context, the inventory surveys only the remaining four languages using *k* = 15 items each. A total of 60 statements are compared in duplicates. In 30 comparisons of two statements, respondents select the statement that corresponds most with their preferred language of appreciation (White, 2016). With internal consistencies between 𝛼 > 0.6 and 𝛼 < 0.8, the scale’s reliability ranges from questionable to acceptable (Blanz, 2023).

Another way to look at appreciation at work is the definition by Döring-Katerkamp and Rohrmeier (2016). Following their understanding, appreciation in the workplace should be a combination of the four distinct facets “respect,” “opportunity,” “self-efficacy,” and “success and recognition.” To measure appreciation, the *German Appreciation Index* can be used (Döring-Katerkamp and Rohrmeier, 2016). This instrument is based on a total of *k* = 14 items, which can be answered on a five-point Likert scale from 1 (*strongly disagree*) to 5 (*strongly agree*). Three items each focus on the dimensions of respect, opportunity, and self-efficacy, while five questions cover the dimension of success and recognition. Three additional questions are implemented to assess experienced appreciation on a global level. With values between 𝛼 > 0.7 and 𝛼 < 0.9, the internal consistency of the instrument is acceptable to high (Blanz, 2023).

Pointing toward the diverging definition and measurement of appreciation, Semmer et al. (2019) assemble appreciation based on the expressions “praise and gratitude,” “trust,” “responsibility,” “support,” and “respect.” Despite these different clusters, Semmer et al. (2019) understand appreciation as a single-faceted construct in distinction to social support. To measure appreciation, the *Appreciation at Work Scale* (AAWS; Jacobshagen et al., 2008) can be used. Five of the *k* = 10 items focus on colleagues, the remaining five on supervisors as the origin of experienced appreciation. Instead of encompassing both conditional and unconditional manifestations, the items focus solely on conditional expressions of appreciation. The items can be answered on a seven-point Likert scale from 1 (*strongly disagree*) to 7 (*strongly agree*). With 𝛼 > 0.8, the instrument’s reliability is high (Blanz, 2023).

Based on the assumption that experienced appreciation should be understood as a one-dimensional construct (Jacobshagen et al., 2008; Resch and Bellhäuser, 2025; Semmer et al., 2019), the two single items by Bakker et al. (2007) and Stocker et al. (2019) do represent another way of surveying appreciation. The former measures the appreciation from colleagues on a five-point rating scale from 1 (*almost never*) to 5 (*very often*). The latter focuses on supervisors as a source of experienced appreciation measured on a seven-point rating scale from 1 (*very dissatisfied*) to 7 (*very satisfied*).

In distinction to other definitions, Mettler von Meibom (2007) as well as Schäfer (2021) focus more explicitly on unconditional manifestations of appreciation. In line with their understanding, appreciation should be understood as a loving and benevolent attitude toward others that is shown by unconditionally acknowledging a person’s worth as a human being. Although their approach is in line with recent findings pointing out the relevance of unconditional appreciation (Döring-Katerkamp and Rohrmeier, 2016), their definition is limited to theoretical considerations without the attempt to operationalize the construct.

Since this section aims to coherently summarize different approaches to define and measure appreciation, Appendix A provides an overview of the most relevant information and exemplary items.

### 2.2 Experienced appreciation in social interactions—an integrative approach

To our knowledge, the previous approaches either lack (1) a well-established theoretical framework, (2) an integrative understanding of appreciation that combines conditional and unconditional manifestations or (3) a robust statistical validation of the assumed construct characteristics.

Considering the outlined definitions, EA-SI aims to combine different understandings of experienced appreciation in one integrative model based on the premises of the *Stress as Offense to Self-theory* (SOS; Semmer et al., 2019). It distinguishes between the *Appreciator,* the person who supposedly sends appreciative signals, and the *Appreciation Receiver,* who feels appreciated. EA-SI puts the feelings of the receiving person in the center of attention. It measures whether the appreciation receiver feels appreciated instead of whether the appreciator intended to send appreciative signals.

An appreciative interaction can be experienced in direct or indirect contact, verbally or non-verbally, and in dyadic or more public interactions (Stocker et al., 2014; Resch and Bellhäuser, 2025). In the work context, colleagues and direct supervisors can be considered sources of experienced appreciation (Stocker et al., 2014).

EA-SI integrates the variety of different behaviors identified as appreciative in the scientific literature in one construct. Döring-Katerkamp and Rohrmeier (2016) pointed out that conditional appreciation is more present in literature than unconditional appreciation. Nonetheless, respondents in the corresponding survey longed the most for precisely this kind of unconditional appreciation regardless of any requirements (Döring-Katerkamp and Rohrmeier, 2016). To recognize these insights, EA-SI integrates both conditional and unconditional expressions of appreciation.

EA-SI is conditionally present when employees feel that their achievements, strengths, and abilities are recognized (Kuoppala et al., 2008; Stocker et al., 2014) and their professional opinions are taken seriously (Stocker et al., 2019). The more trust is placed in them, the stronger the feeling of being appreciated (Elfering et al., 2007). Furthermore, appreciation is higher the more employees receive appropriate material and non-material tokens of acknowledgment for their work (White, 2016).

Focusing on unconditional expressions of EA-SI, the more respondents are treated respectfully (Apostel et al., 2018) and without violence (Rosenberg, 2015), the more they feel appreciated. It is an unconditional expression of EA-SI if employees feel that others are actively listening and showing personal and professional interest (Stocker et al., 2019), are willing to invest time and resources in their well-being (Semmer et al., 2019), and support their development (Siegrist, 2016). Additionally, EA-SI includes the opportunity to emotionally and socially bond with others (Rogers, 2020) and to be part of a community. Conclusively, EA-SI can be described as the integrative combination of conditional and unconditional expressions of a loving and benevolent attitude experienced in social interactions by the Appreciation Receiver (Mettler von Meibom, 2007).

As mentioned above, appreciation can be theoretically distinguished from social support. While social support is described as helpfully interacting with others, appreciation is not limited to helping others but also focuses on acknowledging their worth and performance in all situations of their (work) lives. Following Pfister et al. (2020), social support is present in challenging times while appreciation should be present throughout the highs and lows of people’s work reality. This sets EA-SI apart from social support since it describes appreciating a person’s value in challenging times as well as recognizing their achievements and strengths in times of success.

Based on the integrative definition that considers conditional and unconditional manifestations of appreciation, EA-SI targets one relevant aspect other definitions lacked. A second one is the omission of integrating appreciation in a strong theoretical framework. This is why Resch and Bellhäuser (2025) decided to develop EA-SI upon the well-established Stress as Offense to Self-theory (Semmer et al., 2007, 2019).

The SOS assumes that building and maintaining a positive self-esteem represents a basic human need. If one’s self-esteem is threatened and the corresponding need is jeopardized, individuals strive to defend it. The endeavor to counteract the attack on the self-esteem is accompanied by an increased feeling of stress which is associated with various negative outcomes. Semmer et al. (2019) defined *boosts* and *threats* that influence the self-esteem. Boosts contribute to an increase in positive self-esteem, while threats endanger it. In line with this, employees who felt appreciated at work (boost) reported higher self-esteem and lower stress (Semmer et al., 2019).

Although other theories from organizational psychology, such as the well-established *Conservation of Resources Theory* (COR; Hobfoll et al., 2016), *Job Demands-Resources Theory* (Bakker et al., 2014), or the *Self-Determination Theory* (SDT; Ryan and Deci, 2018) could have been considered the theoretical foundation, we decided to build EA-SI upon the SOS theory.

While the COR distinguishes three categories of resources that must be protected to avoid stress, the JD-R combines resources and demands in a complex interplay with each other. The SDT explicitly focuses on the three human needs “autonomy,” “competence,” and “relatedness” and different outcomes varying with the fulfillment of these needs.

This is where the SOS theory—centered around the individual’s self-esteem—differs from other theories. In line with the theoretical discussion by Semmer et al. (2019), we do not understand the human self-esteem as one of numerous outcomes varying with the complex interplay at work but, on the contrary, as a central construct explaining the mechanism of action behind these interplays. Understanding the lack of appreciation as a severe attack on the self-esteem, we decided to build EA-SI on the SOS theory to recognize the assumed role of appreciation in fulfilling the underlying human need.

For a more detailed theoretical discussion of the SOS theory in contrast to existing theories, see Semmer et al. (2019). By embedding an integratively defined and statistically validated understanding of appreciation—in terms of EA-SI—into the well-established SOS theory, we adhere to the implications by Semmer et al. (2019) that appreciation should be further investigated as an autonomous construct.

The integration of EA-SI in the framework of the SOS theory has been previously tested by Resch and Bellhäuser (2025) in a multi-study design. The results strengthened the assumptions of the SOS theory and the theoretical foundation of the model since EA-SI was positively related to self-esteem, while stress and self-esteem were negatively related to each other. Extending the SOS theory, the results pointed toward direct relations between EA-SI and stress as well as between EA-SI and employee motivation, well-being, and satisfaction (Resch and Bellhäuser, 2025). Exceeding the original SOS theory, these relations were true for employees’ global stress perception and global self-esteem, indicating spillover dynamics between different areas of employees’ lives.

As an extension of existing measures Resch and Bellhäuser (2025) developed the integrative EA-SI Work Scale to operationalize EA-SI and counteract the described incongruence in defining and measuring experienced appreciation at work. The instrument encompasses two scales with *k* = 15 items for colleagues and direct supervisors each. The items can be answered on a 10-point Likert scale from 1 (*strongly disagree*) to 10 (*strongly agree*). The scale also includes the fallback option “I cannot answer this.” With an internal consistency of 𝛼 > 0.9 and a retest-reliability of 𝛼 > 0.8, the reliability for both sources of experienced appreciation is high to excellent (Blanz, 2023). The items are depicted in detail in Appendices B, C.

The instrument was validated in two independent samples with *N* = 231 and *N* = 391 participants. The one-dimensional structure of EA-SI was not solely derived theoretically but statistically tested in exploratory and confirmatory factor analyses (Resch and Bellhäuser, 2025). The assumed relations between EA-SI, employee stress, self-esteem, satisfaction, and emotional exhaustion were found in both samples. The high heterogeneity of participating organizations and branches increased the external validity of the findings. However, regardless of the strengths and practical benefits of the EA-SI Work Scale, the high number of items, high internal consistency, and high correlations with convergent measures require the development of a short scale to facilitate a more time-efficient measurement of EA-SI.

**Figure 1:**
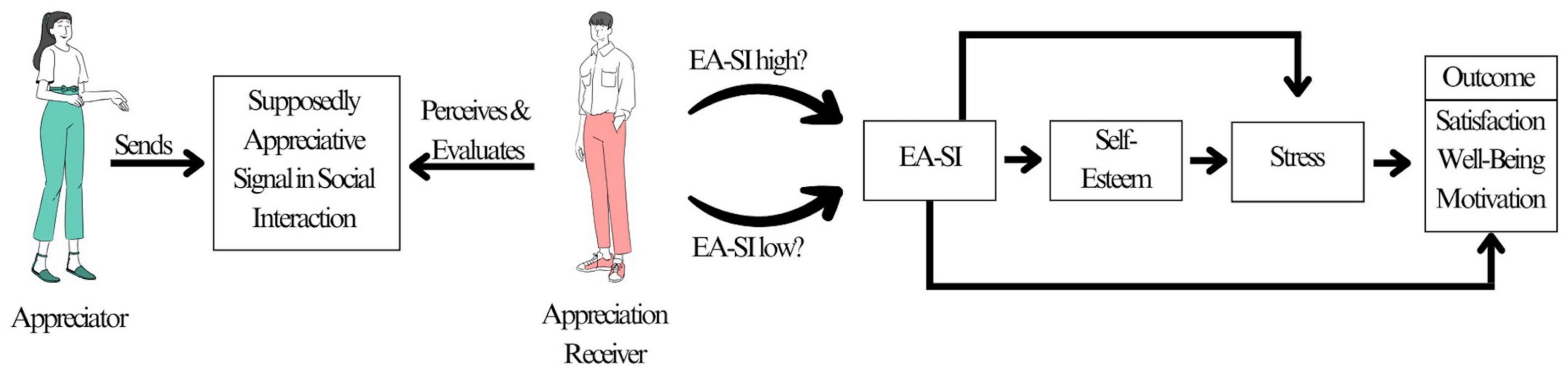
Visualization of the EA-SI model. The model combines EA-SI with the SOS theory and basic considerations of communications.

Despite the various expressions combined in the construct, EA-SI can be understood as a one-dimensional construct. Resch and Bellhäuser (2025) highlighted in their multi-study validation that exploratory and confirmatory factor analyses indicated a one-factor solution. Moreover, EA-SI was statistically distinguishable from social support, incrementally predicting employee motivation, satisfaction, and emotional exhaustion. Figure 1 sums up the EA-SI model.

### 2.3 Research questions and hypotheses

Since EA-SI has already been investigated in two independent samples with the rather time-consuming EA-SI Work Scale, we will develop a more time-economic and low-threshold applicable short scale. To address the described research gap, this article strives to answer three research questions. To increase the article’s stringency, order and wording of the research questions differ from preregistration.

Q1: Does the short version of the EA-SI Work Scale confirm the expected unidimensionality of EA-SI?

Q2: Can EA-SI be measured reliably and validly using the EA-SI Work Scale (short)?

Q3: Is EA-SI related to employees’ work engagement and burnout when controlled by social support?

The following hypotheses, H1 to H3, are derived to replicate the premises of the SOS to test the construct validity of the short scale. The hypothesis supplement “a” refers to colleagues, while supplement “b” refers to direct supervisors as a source of appreciation.

H1a/b: The more employees feel appreciated, the higher their self-esteem.

H2: The higher employees’ self-esteem, the lower their perceived stress.

H3a/b: The more employees feel appreciated, the lower their perceived stress.

As deduced, experienced appreciation—regardless of the incongruence in measuring it—was related to employee work engagement, satisfaction, and well-being (Resch and Bellhäuser, 2025). Emotional exhaustion was used in previous studies to measure well-being. Based on these findings, we assume that EA-SI does not solely relate to one facet of burnout but to the global construct with its three facets “emotional exhaustion,” “loss of meaning,” and “reduced sense of efficacy” (Maslach and Leiter, 2017). Hypotheses H4 and H5 are defined to investigate the criterion validity of the EA-SI Work Scale (short). Social support from colleagues and direct supervisors is implemented as a control variable in these hypotheses to replicate the assumed incremental validity of EA-SI.

H4a/b: The more employees feel appreciated, the higher their work engagement.

H5a/b: The more employees feel appreciated, the lower their burnout.

To answer the deduced research questions and hypotheses, we investigated two independent samples in a multi-study design. Study One was used to develop the EA-SI Work Scale (short) based on the original long version of the instrument. Subsequently, we used Study Two to validate the newly developed EA-SI Work Scale (short) in a second, independent sample.

## 3 Study One

### 3.1 Method—Study One

#### 3.1.1 Design and sample

Study One results from the survey conducted by Resch and Bellhäuser (2025) to validate the original EA-SI Work Scale. The data was surveyed applying a cross-sectional field study design. It was conducted using online self-reports via the service SoSci Survey (Leiner, 2024) available on internal servers of Johannes Gutenberg University Mainz. Small and medium-sized companies across Germany were acquired using Email, flyers, telephone calls, and on-site meetings. The survey initially included two time points, but solely the first measurement point was used to develop the short scale. Data collection took place in the fall of 2022.

There were a total of five inclusion criteria for participation in the survey. Participants had to (1) be between 18 and 79 years old, (2) work at least 8 h a week, and (3) have regular contact with at least one colleague and direct supervisor. In addition, only those participants who (4) completed the questionnaire to the last page and (5) stated at the end that they had answered it conscientiously and concentrated were considered.

This resulted in a total sample of *N* = 391 employees. *n* = 260 (66.5%) were female, *n* = 129 (33%) were male, and *n* = 2 (0.5%) reported their gender to be non-binary. On average, participants were 34.04 years old (*SD* = 13.67). The youngest participant was 18, while the oldest one was 71 years old. Overall, 51% of respondents were in permanent employment, 34.5% were employed temporarily, 12.8% worked in the public sector, and 1.7% worked as freelancers. The sample is characterized by a highly heterogeneous composition of different professional activities. In addition to doctors, participants included teachers, graphic designers, cooks, service personnel, architects, caregivers, and various other branches.

#### 3.1.2 Instruments

To measure experienced appreciation, the previously described EA-SI Work Scale by Resch and Bellhäuser (2025) was used. In the instructions, participants were asked to remember the last 3 months when answering the questionnaire. In addition to experienced appreciation, various other constructs were surveyed to investigate employee well-being, satisfaction, and motivation. However, these instruments are of no further relevance to deriving the EA-SI Work Scale (short).

#### 3.1.3 Transparency and openness

All relevant instruments and inclusion criteria are described. The processed data from which the EA-SI Work Scale (short) was derived can be accessed publicly via osf.io using the link https://osf.io/hyk2m/?view_only=21c425cdcf294ad39e399a7b554c4d08. As the data analysis was performed with JASP 0.18.3 (JASP Team, 2024), no code was published. Due to the fallback option within the EA-SI Work Scale, missing values occurred. Since the proportion of missing values (4.4%) was below the recommended threshold of 10%, missing values were estimated using mean imputation (Schumacker, 2015; Watkins, 2018). The research questions and hypotheses were preregistered using aspredicted.org^1^.

#### 3.1.4 Statistical procedure—development of the EA-SI work scale (short)

To derive the short scale and analyze the construct’s dimensional structure, the original EA-SI Work Scale was analyzed using confirmatory factor analyses for both sources. To determine the factor structure of the original scale, the maximum likelihood method with 5,000 bootstrap samples and standardized factor loadings was applied. Since the factorial structure of the original scale has already been tested by Resch and Bellhäuser (2025), the data in Study One was solely used to derive the short scale.

To do so, we defined specific criteria to guarantee a comprehensible and reliable item selection. In the first step, (1) only the items with an “excellent” factor loading ≥0.71 (Comrey and Lee, 1992) were selected for the short scale. Based on the original scale’s length of *k* = 15 items for each source of appreciation, the aim was to reduce the short scale to a maximum of one-third of its original length. Hence, (2) the maximum number of items in the more time-efficient short scale should not exceed *k* = 5. We then (3) implemented a data-driven interim phase of item pre-selection using artificial intelligence (AI) to perform a first draw of items based on the construct definition and the previously defined criteria. Using a specific prompt, we asked ChatGPT 4.0 (Open AI, 2024) to select a restricted number of items that survived confirmatory analysis. Within several runs, the AI selected different constellations for both sources of appreciation. In line with the guidelines for developing instruments with high psychometric quality (Moosbrugger and Kelava, 2008), the remaining items were also presented to naïve and expert judges. Those judges evaluated the items’ content validity, considering the construct’s definition and the defined selection criteria. The instruction and the specific selection criteria for AI’s and experts’ decisions can be found in Appendix D. Finally, the authors combined all information to decide which items to include into the short scale. Please note that the factor structure of the original scale has been previously tested by Resch and Bellhäuser (2025) and that these analyses are reported again for comprehensibility and integrity in Study One.

### 3.2 Results

#### 3.2.1 Factor structure—original EA-SI work scale

Considering all indices and the misfit plots, the results indicated a good fit for the single-factor solution for colleagues and direct supervisors. The parameters of the analyses are shown in Table 1. The misfit plots are depicted in Appendix E.

**Table 1:** Confirmatory factor analyses – original EA-SI work scale.

Indices	Colleagues	Supervisors
χ^2^	180.65	231.66
df	90	90
p	<0.001	<0.001
CFI	0.97	0.97
RMSEA	0.05	0.06
90% CI	[0.04; 0.06]	[0.05; 0.07]
SRMR	0.03	0.03

In addition to the fit indices, the 90% confidence interval of the RMSEA (90% CI), the significance value of the χ^2^ test (p), and the degrees of freedom (df) are reported.

#### 3.2.2 Item selection—EA-SI work scale (short)

The items for the short scale were selected according to the defined criteria. First, all items with a factor loading <0.71 were excluded. This step left *k* = 9 items for both sources.

The remaining nine items—separated by source—were then fed into the AI. The AI made several pre-selections based on the prompt using the construct definition to fulfill the task of reducing the instrument to one-third of its original length. Simultaneously, *n* = 18 naïve and *n* = 10 expert judges assessed the items. Combining the factor analyses, AI’s pre-selection, and judges’ assessment, we selected *k* = 4 items. The same items with the identifiers 10, 5, 2, and 11 were selected for colleagues and direct supervisors. Table 2 summarizes the factor loadings of all items of the original EA-SI Work Scale.

**Table 2:** Factor loadings—original EA-SI work scale.

Colleagues			Supervisors		
Factor	Item	Loading	Factor	Item	Loading
Factor 1	EA-SIc_10	0.86	Factor 1	EA-SIc_10	0.92
	EA-SIc_5	0.85		EA-SIc_5	0.92
	EA-SIc_2	0.83		EA-SIc_12	0.91
	EA-SIc_6	0.81		EA-SIc_2	0.90
	EA-SIc_11	0.77		EA-SIc_6	0.87
	EA-SIc_14	0.74		EA-SIc_11	0.85
	EA-SIc_4	0.72		EA-SIc_15	0.79
	EA-SIc_8	0.71		EA-SIc_1	0.78
	EA-SIc_1	0.71		EA-SIc_4	0.73
	EA-SIc_12	0.58		EA-SIc_14	0.66
	EA-SIc_15	0.54		EA-SIc_13	0.61
	EA-SIc_13	0.49		EA-SIc_8	0.58
	EA-SIc_3	0.44		EA-SIc_3	0.39
	EA-SIc_7	0.27		EA-SIc_9	0.34
	EA-SIc_9	0.27		EA-SIc_7	0.30

The left section shows the standardized factor loadings for colleagues, while the right section shows them for direct supervisors.

The short scale was highly correlated with the original EA-SI Work Scale (Cohen, 1988). For colleagues, the correlation was *r* = 0.92. For direct supervisors, both instruments correlated with *r* = 0.95. For a detailed list of the items included in the EA-SI Work Scale (short), see Appendices F, G.

#### 3.2.3 Internal consistency

The internal consistency was excellent for the original EA-SI Work Scale 𝛼 = 0.90 (colleagues), 𝛼 = 0.93 (direct supervisors), as well as for the EA-SI Work Scale (short) 𝛼 = 0.89 (colleagues), 𝛼 = 0.94 (direct supervisors; Blanz, 2023).

## 4 Study Two

After developing the EA-SI Work Scale (short), the instrument was validated in a second, independent sample. The following section will outline Study Two’s design, sample, instruments, and statistical analyses.

### 4.1 Method—Study Two

#### 4.1.1 Design and sample

The second sample was collected using a cross-sectional field study design to validate the short scale. In total, *N* = 323 participants were surveyed. The inclusion criteria, acquisition strategy, and method of online-based self-reports equal Study One. Data collection took place in the fall of 2023.

Of the participants, *n* = 238 were female, *n* = 83 were male, and *n* = 1 were non-binary. The age ranged from 18 to 64 years (*M* = 32, *SD* = 12.24). Of all respondents, 57.3% were in permanent employment, 32.2% were in temporary employment, 8.4% were civil servants, and 2.2% worked as freelancers. The sample was characterized by high job heterogeneity. Participants include tax consultants, police officers, physiotherapists, leading personnel, logopaedists, service personnel, laboratory technicians, church personnel, and teachers.

#### 4.1.2 Instruments

EA-SI was measured using the newly developed EA-SI Work Scale (short). In line with the original EA-SI work Scale, a ten-point Likert scale was used. Since appreciation should be a highly nuanced experience, we decided to provide respondents with a scale that mirrors the needed degree of distinction based on its highly granulated answer options (Dawes, 2008). Moreover, the lack of a neutral answer option should help respondents to decide whether they would rather agree or disagree with a specific statement instead of falling for neutrality (Dawes, 2008). Therefore, the items—four for colleagues and four for direct supervisors as a source of appreciation—could be rated from 1 (*strongly disagree*) to 10 (*strongly agree*). If the items did not apply to their work, participants could use the fallback option “I cannot answer this.” Participants were instructed to remember the last 3 months when answering the questionnaire.

To determine the short scale’s convergent validity, we investigated its relation with the previously described single items to measure appreciation by direct supervisors (Stocker et al., 2019) and colleagues (Bakker et al., 2007). The first single item ranged from 1 (*almost never*) to 5 (*very often*). The second item could be answered on a seven-point rating scale from 1 (*very dissatisfied*) to 7 (*very satisfied*).

To test for discriminant validity, we used four established items to survey participants’ attitudes toward the government’s environmental policy (Ferris et al., 2008). The question for all four items was, “How do you rate our government’s environmental policy?.” The items were answered on a five-point dyadic differential from 1 (*good*/ *wise*/ *helpful*/ *useful*) to 5 (*bad/dumb/damaging/useless*).

Furthermore, employees’ global self-esteem was measured using the German version of the *Rosenberg Self-Esteem Scale* (von Collani and Herzberg, 2003). The scale contained 10 items on a six-point Likert scale from 1 (*not at all*) to 6 (*completely*). An example item is “Overall, I am satisfied with myself.”

As an additional convergent measure, we surveyed *perceived stress* with the German version of the *Perceived Stress Questionnaire* (Fliege et al., 2001) using the short version by Umucu et al. (2018). The eight items could be answered on a four-point rating scale from 1 (*almost never*) to 4 (*most of the time*). One item read: “You have the feeling that too many demands are asked of you.”

*Work engagement* was measured using the German version of the Utrecht Work Engagement Scale-9 by Schaufeli et al. (2006). The scale has nine items that can be answered on a seven-point rating scale from 1 (*never*) to 7 (*every day*). An example item is “I am full of exuberant energy at work.”

To assess *burnout,* the short version of the Maslach Burnout Inventory for Students (MBI-SS KV) by Wörfel et al. (2015) was used. For Study Two, the items were adapted to fit the work context. For example, the item “My studies make me feel drained” was changed to “My work makes me feel drained.” The scale covers all three dimensions of burnout with three items each. The items were answered on a seven-point rating scale from 1 (*never*) to 7 (*every day*).

Social support was implemented as a control variable to analyze the incremental validity of the EA-SI Work Scale (short). To do so, we used the previously described *Social Support Scale* by Frese (1989).

In addition to these instruments, demographic data was collected, including gender, age, educational qualification, current professional activity, student status, contractually agreed and actual working hours, and job title. Moreover, the turnover intention was collected. This measure is of no interest to this article.

#### 4.1.3 Transparency and openness

We described all instruments and the inclusion criteria of Study Two. The data of the validation of the EA-SI Work Scale (short) is publicly available via osf.io using the link https://aspredicted.org/bwdr-pwng.pdf. The data were analyzed using JASP 0.18.3 (JASP Team, 2024), which is why no code was published. The fallback option in the EA-SI Work Scale (short) resulted in missing values. The proportion of missing data (0.55%) was less than 10%, so missing values were imputed by mean imputation (Schumacker, 2015; Watkins, 2018). The research questions and the hypotheses of this article were preregistered at aspredicted.org (see footnote 1). While the three sub-facets of burnout were listed separately in the pre-registration, we decided to examine burnout as a global construct for reasons of stringency. Moreover, we decided to change the research questions in terms of wording but not in terms of content to enhance the stringency of the article in line with the review process. Please note that in the original raw data, there were *k* = 7 items for each source of EA-SI as the survey was carried out as part of a Master’s thesis that made use of these items. Nonetheless, for the sake of comprehensibility, the processed data includes the described EA-SI Work Scale (short) with *k* = 4 items only. All following analyses are built upon the uploaded scale and constructs.

#### 4.1.4 Statistical procedure—validation of the EA-SI work scale (short)

In the first step, the aim was to replicate the assumed one-dimensional structure of EA-SI in Study Two. To do so, we conducted confirmatory factor analyses using the maximum likelihood rotation with 5,000 bootstrap samples.

The χ^2^-test was computed to assess the overall model fit. A significant χ^2^-Test points toward a low model fit (Hu and Bentler, 1999; Xia and Yang, 2019). However, when interpreting χ^2^, it should be noted that the risk of a ß-error – the erroneous rejection of a suitable model – increases with sample sizes above *N* = 200 (Bergh, 2015; Bühner, 2021; Yukl et al., 2002).

Consequently, Hu and Bentler (1999) recommended considering additional parameters when determining the model fit. For this reason, the indices *Comparative Fit Index* (CFI), *Root Mean Square Error of Approximation* (RMSEA), and *Standardised Root Mean Square Residual* (SRMR) were calculated to determine the fit of the one-dimensional solution. These indices are less sensitive to the sample size (Bühner, 2021). Values of RMSEA <0.08 (Awang, 2012), SRMR <0.08 (Byrne, 2013), and CFI > 0.96 (Hu and Bentler, 1999) indicate a good fit. While the RMSEA is sensitive to the number of degrees of freedom—erroneously rejecting fitting models with decreasing degrees of freedom (Chen et al., 2008; MacCallum et al., 1996)—the CFI and SRMR are robust against sample size and degrees of freedom.

As recommended by Kenny et al. (2015), in addition to the χ^2^-test, the misfit plots were calculated to determine the global fit of the model. These plots test the dyadic error variance between the individual items of the scale. High values indicate a low model fit (Kenny et al., 2015). Rogers (2024) suggested that a low “misfit” is indicated if the majority of values lies below *r* = 0.10.

In the second step, Pearson product–moment correlations were calculated to determine the short scale’s construct validity. Convergent validity was indicated when the short scale correlated positively with theoretically similar constructs and negatively with distinct constructs. Discriminant validity was indicated when EA-SI was not correlated with discriminant measures Döring and Bortz, 2016.

Finally, the criterion validity of the short scale was analyzed. Pearson product–moment correlations were used to replicate the relations assumed based on the SOS. To test for EA-SI’s incremental validity, hierarchical regression analyses were conducted using social support as a control variable. First, social support was included in the model, and then EA-SI was included as a predictor. Participants’ work engagement and burnout served as criteria. The significance level for all analyses was *p* < 0.05 (Fisher, 1997).

### 4.2 Results

#### 4.2.1 Factorial validity—EA-SI work scale (short)

Confirmatory factor analyses were calculated using the four selected items of the short scale separated by colleagues and direct supervisors as sources of experienced appreciation. The χ^2^-test was not significant for colleagues, indicating a good model fit. For direct supervisors, however, the χ^2^-test was significant. In contrast, the additionally computed misfit plots indicated an excellent model fit with *r* < *0*.10 for all items and both sources. CFI and SRMR supported this assumption of an excellent fit of the one-dimensional solution. The RMSEA provided ambiguous results. Table 3 summarizes the results of the CFA. The misfit Plots are depicted in Appendix H.

**Table 3:** Confirmatory factor analysis – EA-SI work scale (short).

Parameters	Colleagues	Direct supervisors
χ^2^	5.09	20.76
df	2	2
*p*	0.078	<0.001
CFI	0.99	0.99
RMSEA	0.07	0.17
90% CI	[0.00; 0.15]	[0.11; 0.24]
SRMR	0.01	0.01

In addition to the fit indices, the 90% confidence interval of the RMSEA (90% CI), the significance value of the χ^2^ test (p), and the degrees of freedom (df) are reported.

#### 4.2.2 Construct validity—EA-SI work scale (short)

As expected, the short scale was positively related to the convergent single item for colleagues (*r* = 0.72^**^, p < 0.001) and direct supervisors (*r* = 0.88^**^, p < 0.001). There was no significant correlation between EA-SI and employees’ attitudes toward environmental politics (colleagues: *r* = 0.07, *p* = 0.211; direct supervisors: *r* = −0.05, *p* = 0.375), supporting the discriminant validity of the short scale. Consequently, the results support the construct validity of the EA-SI Work Scale (short).

#### 4.2.3 Criterion validity—EA-SI work scale (short)

In line with the hypotheses, employees’ self-esteem was positively related to experienced appreciation from colleagues *r* = 0.32^**^, p < 0.001, and direct supervisors *r* = 0.25^**^, p < 0.001. Employees who felt less appreciated by their colleagues and direct supervisors reported higher levels of stress (colleagues *r* = −0.33^**^, p < 0.001; direct supervisors *r* = −0.24^**^, p < 0.001). Self-esteem was negatively related to employee stress perception *r* = −0.48^**^, *p* < 0.001. Therefore, the assumptions of the SOS could be replicated, supporting the hypotheses one to three. A detailed depiction of the internal consistency, mean, standard deviation, range, and inter-correlations of all constructs can be found in Appendix I.

In the second step, linear hierarchical regressions were conducted using social support and EA-SI as predictors. As expected, EA-SI from colleagues predicted higher work engagement (*β* = 0.41, *t* = 6.33, *p* < 0.001) above social support (*β* = −0.01, *t* = −0.18, *p* = 0.860) with *R*^2^ = 0.16, *F*(2,320) = 30.88, *p* < 0.001. Experienced appreciation from direct supervisors did also incrementally contributed to the prediction of work engagement (*β* = 0.33, *t* = 4.13, *p* < 0.001) after social support was added (*β* = 0.05, *t* = 0.57, *p* = 0.57), *R*^2^ = 0.14, *F(2*,320) = 25.29, *p* < 0.001.

In line with the hypotheses, EA-SI predicted burnout beyond social support. The more employees felt appreciated by their colleagues, the lower their burnout (*β* = −0.32, *t* = −4.92, *p* < 0.001), above social support (*β* = −0.08, *t* = −1.24, *p* = 0.215), *R*^2^ = 0.14, *F*(2,320) = 26.47, *p* < 0.001. The higher EA-SI by direct supervisors the lower employee burnout (*β* = −0.28, *t* = −3.58, *p* < 0.001) beyond social support (*β* = −0.19, *t* = −2.52, *p* = 0.012), with *R*^2^ = 0.19, *F*(2,320) = 39.53, *p* < 0.001. Hence, the findings did support hypotheses four and five.

## 5 Discussion

This article pointed toward the incongruency in understanding appreciation, gave a detailed overview of how appreciation is defined and measured in literature, and introduced the construct EA-SI as an integrative alternative. In addition, this article aimed to (1) test the unidimensional structure of EA-SI based on the EA-SI Work Scale (short), (2) validate the EA-SI Work Scale (short) while replicating the assumptions of the SOS theory, and (3) investigate the role of appreciation for employee work engagement and burnout above the influence of social support. The following section will discuss the results of Study One and Study Two against theory and previous empirical findings. The article’s limitations will be critically reviewed, and implications for future research and practical use will be derived.

### 5.1 Internal consistency

The internal consistency of the short scale was 𝛼 = 0.88 for colleagues and 𝛼 = 0.96 for supervisors. Therefore, its reliability can be evaluated as high to excellent (Blanz, 2023).

### 5.2 Factorial validity

Using two independent samples, the short scale was developed based on the original EA-SI Work Scale and then validated in the second sample. In the first sample, the χ^2^-test was significant for colleagues and direct supervisors. In the second sample, the χ^2^-test indicated a good fit of the single-factor model for colleagues but not for direct supervisors.

The RMSEA indicated an acceptable to good fit for the original EA-SI Work Scale. In Study Two, the RMSEA supported the one-dimensional structure for colleagues but not for direct supervisors (Hu and Bentler, 1999). When interpreting the RMSEA, it must be considered that Study One and Study Two differed substantially regarding their degrees of freedom. The lower the degrees of freedom, the higher the tendency of the RMSEA to reject fitting models (Kenny et al., 2015; MacCallum et al., 1996).

In contrast, the CFI and SRMR suggested an excellent fit of the one-dimensional solution for colleagues and direct supervisors in both studies (Hu and Bentler, 1999). The misfit plots also pointed toward a good to excellent fit of the overall single-factorial model for colleagues and direct supervisors (Rogers, 2024).

Considering our findings and the limitations of the tested parameters, the one-factorial solution is supported in both studies. Thus, the first research question (Q1) can be answered: Using the EA-SI Work Scale (short) the unidimensional structure already found for the long version was strengthened.

### 5.3 Construct validity

EA-SI was positively related to both single items to measure appreciation. In turn, the EA-SI Work Scale (short) was unrelated to employees’ political opinions. Hence, the results of the Pearson product–moment correlations point toward the short scale’s convergent and discriminant validity.

### 5.4 Criterion validity

The more employees felt appreciated by their colleagues and direct supervisors, the higher their self-esteem and the lower their perceived stress. The higher employees’ self-esteem, the lower their perception of stress. Therefore, the results of Study Two supported hypotheses one to three for both sources of appreciation. Hence, the presumptions of the SOS theory and the expected relations were replicable in an independent sample.

#### 5.4.1 Incremental validity

As expected, higher levels of EA-SI were related to higher employee work engagement and lower burnout. This was true for colleagues and direct supervisors as sources of experienced appreciation. In all hierarchical regression analyses, EA-SI predicted the criteria above social support. Moreover, in most analyses, EA-SI remained the only significant predictor, while social support did not provide an incremental contribution that exceeded the predictive value of experienced appreciation. The explained variance was moderate, varying between *R^2^* = 0.14 and *R^2^* = 0.19 (Cohen, 1988). Accordingly, hypotheses four and five can be accepted.

Considering the short scale’s content, construct, and criterion validity as well as its excellent internal consistency, we can answer the second research question (Q2): The construct “Experienced Appreciation in Social Interactions” can be reliably and validly measured using the newly developed EA-SI Work Scale (short).

Moreover, EA-SI predicted employees’ work engagement and burnout beyond social support. These findings point toward the relevance of appreciation for employee burnout and work engagement. Therefore, research question three (Q3) can be answered. The results strengthen the approach to understand experienced appreciation as an autonomous construct that is distinguishable from other constructs such as social support. Since, in most of the analyses, social support was ruled out as a predictor after EA-SI was added, the question is raised whether social support should be understood as an expression of appreciation instead of vice versa.

### 5.5 Limitations and theoretical implications

Besides the outlined strengths, some limitations should be addressed, raising new questions that cannot be answered within the scope of this article. First, it should be noted that the validation of the EA-SI Work Scale (short) is limited to cross-sectional self-reports. Future research should analyze EA-SI using different designs to increase the validity of the construct. For example, observation studies and qualitative surveys could be conducted to increase the ecological validity. To increase the internal validity, experiments could be designed to identify specific markers (e.g., facial expressions, gestures, statements) that are perceived as indicators of appreciative behavior.

Although the findings support the construct definition and the theoretical assumptions of the SOS, the different parts of the model were analyzed separately. Diary studies with time-lagged panel data using multi-level analyses and mediated mediation analyses should be conducted to better understand EA-SI’s mechanism of action and validate the assumed direction of the relations within the EA-SI model.

Considering the RMSEA’s contradictory results, the factorial structure of EA-SI—even if confirmed in different independent samples—should be investigated in samples with more degrees of freedom or participants. Besides the small number of degrees of freedom, there is an uneven distribution of gender and terms of employment. Future research should examine the replicability of the results in samples with various gender ratios and more widely distributed employment relationships.

In addition, using the data-based pre-selection of items by the AI “ChatGPT” as a decisional criterion in scale construction represents an innovative approach. Nevertheless, the suitability of AI within item selection must be further investigated.

Across different studies and based on the results of the current article, the role of experienced appreciation for employee satisfaction, motivation, and well-being implies the development of training programs to foster experienced appreciation at work intentionally. However, to systematically evaluate the effectiveness of such training programs, the scale’s sensitivity to change and the construct’s short-term adaptability must be evaluated in longitudinal designs.

The introductory summary strives to give a fundamental overview of construct definitions and operationalizations of experienced appreciation to clarify the confusing multitude of approaches in the scientific literature. Nonetheless, this article neither represents a systematic review nor a meta-analysis. Future research should systematically entangle the current research landscape around experienced appreciation to enable a more criteria-based clarification of the current state of research around experienced appreciation.

The EA-SI model is based on fundamental assumptions of interpersonal communication. Assuming that (1) the Appreciator and the Appreciation Receiver cannot not communicate at any time (Watzlawick et al., 2016) and that (2) every supposedly appreciative signal must be interpreted by the Appreciation Receiver (Schulz von Thun, 1981) the question arises how specific attitudes and personality traits of the receiver influence EA-SI. Hence, future research should investigate the influence of such moderator variables on the relations between EA-SI and dependent variables.

Based on the findings by Ashforth and Schinoff (2016) and Oyserman et al. (2012), EA-SI was first validated at work due to the identity-shaping relevance of this life area. Nevertheless, EA-SI is not limited to the work context theoretically. Following its definition, EA-SI describes the appreciation individuals experience when interacting with others regardless of the context. Accordingly, current findings indicate the relevance of experiencing appreciation in other areas of individuals’ lives.

For example, Carstensen et al. (2021) showed that students who felt appreciated by their lecturers were more satisfied, more enthusiastic, and less emotionally exhausted in their studies. An appreciative relationship between teachers and students is not only associated with a positive cognitive development of the students (Vandenbroucke et al., 2018) but also with less emotional exhaustion and increased enthusiasm on teachers’ side (Cui, 2022). Moreover, experienced appreciation also matters in close relationships. The more participants in romantic relationships felt appreciated by their partner, the more emotionally attentive they acted, the more willing they were to respond to their partner’s needs, and the less they were willing to end their relationship (Gordon et al., 2012; Gordon and Diamond, 2023).

Nonetheless, these findings are based on incongruent definitions and operationalizations of the construct experienced appreciation. Therefore, the EA-SI Work Scale (short) should be adapted to congruently and comparably examine Experienced Appreciation in Social Interactions in other types of relationships. In addition, the EA-SI Work Scale (short) has been validated in two samples with German employees. Hence, besides the transfer to other types of relationships, the scale’s validation for other languages and cultures is pending.

This article highlights that the constructs EA-SI and social support are distinguishable from each other. This contradicts the understanding of appreciation as a part of social support. Nonetheless, our work is limited to social support and does not focus on other constructs with which appreciation is intermingled. Future research should strive to investigate and entangle supposedly intertwined constructs to clarify them theoretically and statistically.

### 5.6 Practical implications

The EA-SI Work Scale (short) was developed as a time-efficient instrument to operationalize experienced appreciation. This valid and reliable alternative to the original EA-SI Work Scale is primarily intended for scientific purposes. The short scale can be used to analyze experienced appreciation as a theoretically elaborated and statistically validated construct that can be compared between different studies. At the same time, the results did not solely highlight the validity of the short scale but also the practical relevance of EA-SI.

Consequently, in addition to the original EA-SI Work Scale, the comparatively time-economic short scale can be used in organizations to measure whether employees feel appreciated by their colleagues and direct supervisors. The scale’s evaluation is designed to be comprehensible and easily applicable. Therefore, employees’ answers will be aggregated in one (mean) value. Depending on the application area and the issue, organizations must decide whether to use the more information-rich EA-SI Work Scale or the shorter version.

The results can then be used to identify areas of development within the organization. Additionally, the single items of the scale can be interpreted descriptively to identify specific expressions of appreciation that are perceived as poorly expressed. By focusing on these individual expressions of experienced appreciation, the scale offers the opportunity for *Appreciators* and *Appreciation Receivers* to reflect on appreciation. Appreciators can reflect on how they can express appreciation – where missing – less ambiguously and more frequently. Appreciation Receivers could identify their preferences regarding appreciation, learn how to communicate their needs, and become more attentive to certain expressions of experienced appreciation.

However, to achieve these benefits, training programs must be developed based on the EA-SI Work Scale (short), which guide employees through the reflection process and enable them to interpret their results and derive implications from them. Applying the theoretical model of Experienced Appreciation in Social Interactions, we suggest not solely training the Appreciator but also the Appreciation Receiver. However, such programs are still pending. Therefore, we invite science and industry to collaborate in engineering and evaluating training programs to foster an appreciative, positive culture at work built on mindful interaction.

## 6 Conclusion

By pointing out different ways to understand experienced appreciation, this article provides an orienting overview that other works omitted to give. The results of this article point toward EA-SI as a reliable, valid, and unidimensional alternative to the described incongruency in literature. The theoretical assumptions of the SOS theory could be replicated using Study Two as an independent sample. With the development of the EA-SI Work Scale (short), this article contributes to measuring experienced appreciation more time-economically and comparably.

The construct’s definition and the assumed single-dimensional structure were deduced from the literature and investigated empirically. The presumptions of the SOS theory as a foundation of EA-SI were not taken for granted but were statistically investigated to counter the replication crisis (Open Science Collaboration, 2015). The role of experienced appreciation from colleagues and direct supervisors regarding employee motivation and well-being was clarified further.

The branch heterogeneity and professional diversity in both samples suggest high generalizability of the results. Therefore, we expect our work and the short scale to apply to various contexts, leading to a more consistent definition and comparable analyses of experienced appreciation at work.

Nonetheless, our research came across several limitations, producing new questions. Future research should target these limitations to further test and develop the understanding and operationalization of the construct “Experienced Appreciation in Social Interactions.”

## Data Availability

The datasets presented in this study can be found in online repositories. The names of the repository/repositories and accession number(s) can be found at: https://osf.io/hyk2m/?view_only=21c425cdcf294ad39e399a7b554c4d08.

## Footnotes

^1^ https://aspredicted.org/LM6_7C5
