# Corrigendum: Measuring appreciation made EA-SI-the development of a short scale to measure experienced appreciation in social interactions at work

**DOI:** 10.3389/fpsyg.2025.1607593

**Published:** 2025-04-30

**Authors:** Maximilian Stefan Resch, Elena Nagelmann, Henrik Bellhäuser

**Affiliations:** Institute of Psychology, Johannes Gutenberg University Mainz, Mainz, Germany

**Keywords:** appreciation, EA-SI work scale, work engagement, burnout, positive work

In the published article, there was an error in **Supplementary Table 1**, 8.1 Appendix A. Different Approaches to Define and Measure Appreciation. The exemplary items and citations regarding two of the different understandings of appreciation mentioned in Appendix A were erroneous and/or incomplete.

The erroneous items were:

(1) I feel that my work is being rewarded.

(2) I feel satisfied with the rewards I receive for my work.

(3) My salary is adequate for the work I do.

and

(1) I feel that my efforts at work are often recognized and appreciated.

(2) When I do something well, I am praised or thanked for it.

(3) My contributions to the team are valued by my colleagues and supervisor.

The incomplete citation was:

Leiter and Maslach (1999)

Bregenzer et al. (2022)

The erroneous citation was:

White (2016)

The corrected Supplementary Table 1 is published online.

The authors apologize for this error and state that this does not change the scientific conclusions of the article in any way. The original article has been updated.

