# Supplementary material for: Measuring appreciation made EA-SI-the development of a short scale to measure experienced appreciation in social interactions at work
Source: Front Psychol. 2025 Mar 24;16:1465512. doi: 10.3389/fpsyg.2025.1465512 (PMC11973763; doi:10.3389/fpsyg.2025.1465512)
Supplement: Supplementary file 1 [file Table_1.pdf]

# 8 Appendices

## 8.1 Appendix A – Different Approaches to Define and Measure Appreciation

| Construct Name | Definition | Dimensions | Operationalization | Number of Items | Scale | Examplary Items | Authors |
|---|---|---|---|---|---|---|---|
| Respect | "an attitude towards other people […] that in return engenders in the target a feeling of being appreciated" (p. 345) | 1 | Respectful Leadership Scale | 12 | Five-point Likert Scale<br>1 (*not at all*)<br>5 (*very much*) | The leader<br>(1) … expresses criticism in an objective and constructive way.<br>(2) … recognizes me as a full-fledged counterpart.<br>(3) … provides me with any information that is relevant to me. | van Quaquebeke and Eckloff (2010), p.345 |
| Reward | "[…] collegial or managerial recognition, material compensation and intrinsic enjoyment of the work." (p. 110) | 1 | Areas of Worklife Scale | 4 | Five-point Likert Scale<br>1 (*strongly disagree*)<br>5 (*strongly agree*) | (1) I receive recognition from others for my work. | Leiter and Maslach (1999) cited according to Bregenzer et al. (2022), pp.110, 113 |
| Social Support | "[…] overall levels of helpful social interaction available on the job from both co-workers and supervisors" (p.69) | 1 | Social Support Scale | 20 | Four-point Rating Scale<br>1 (*not at all*)<br>4 (*completely*) | (1) How much can you rely on your colleagues/ direct supervisor when things get difficult at work?<br>(2) How willing are your colleagues/ direct supervisor to listen to your work-related problems?<br>(3) How much do your colleagues/ direct supervisor support you so that you have an easier time at work? | Frese (1989)<br>Karasek & Theorell (1990), p.69 |
| Five Languages of Appreciation | Appreciation as the combination of the five dimensions (1) words of affirmation, (2) quality time, (3) acts of support, (4) material gifts, and (5) physical touch | 5 | Motivating by Appreciation Inventory | 60 | Dyadic statement selection | (1) Go to lunch together to talk about business issues.<br>(2) Go to lunch together and not talk about business issues.<br>(3) Have a weekly "check in" meeting to see how things are going. | Chapman & White (2011) accessed via https://mbainventory.com/free-resources/ |
| Appreciation | Appreciation as the combination of the four dimensions (1) respect, (2) opportunity, (3) self-efficacy, and (4) success and recognition. | 4 | German Appreciation Index | 14 | Five-point Likert Scale<br>1 (*strongly disagree*)<br>5 (*strongly agree*) | (1) my direct manager respects me as a competent discussion partner in my area of responsibility<br>(2) I am given the freedom to implement my own ideas if these ultimately also benefit the company.<br>(3) I can develop professionally and personally in my company. | Döring-Katerkamp & Rohrmeier (2016) |
| Appreciation | Appreciation as a combination of the expressions "praise and gratitude", "trust", "responsibility", "support", and "respect" in one single-dimensional construct. | 1 | Appreciation at Work Scale | 10 | Seven-point Likert Scale<br>1 (*strongly disagree*)<br>7 (*strongly agree*) | (1) My supervisor praises me when I do my tasks well.<br>(2) I can express my own opinion in discussions with my supervisor.<br>(3) When my colleagues get into difficult situations and I help them with advice and support, they appreciate it. | Jacobshagen et al. (2008) |
| Appreciation | Appreciation as a single-dimensional construct. | 1 | Single Item | 1 | Seven-point Rating Scale<br>1 (*extremely unsatisfied*)<br>7 (*extremely unsatisfied*) | (1) Overall, how satisfied are you with the appreciation of your person shown by your line manager? | Stocker (2019) |
| Appreciation | Appreciation as a single-dimensional construct. | 1 | Single Item | 1 | Five-point Rating Scale<br>1 (*hardly ever*)<br>5 (*very often*) | (1) Do your colleagues appreciate your work? | Bakker et al. (2007) |
| Appreciation | The loving and benevolent attitude towards others that is transported through likewise behaviour | | Theoretical Approach | / | / | / | Mettler von Meibom (2007)<br>Schäfer (2019) |

## 8.2 Appendix B – Original EA-SI Work Scale – Colleagues

The following questions relate to the appreciation you experience from others in your day-to-day work. The important thing here is that it is about how much you personally feel valued. There are no right or wrong answers. Please answer honestly and conscientiously.

At this point, we are interested in your personal feelings and assessments. Below you will be presented with statements that relate to your colleagues.

*Please indicate to what extent these statements apply to you personally. Remember specifically the last three months. Please tick the option "I cannot answer." only if it does not apply at all to your professional activity or cannot be answered.*

| | |
|---|---|
| EA-SIc_1 | My colleagues take my objections seriously. |
| EA-SIc_2 | My colleagues show me that they know and appreciate my strengths. |
| EA-SIc_3 | My colleagues often entrust me with tasks that are important for the entire team. |
| EA-SIc_4 | My colleagues always emphasise that they can rely on me. |
| EA-SIc_5 | I can clearly feel that my colleagues appreciate my work. |
| EA-SIc_6 | I feel seen by my colleagues with all my successes. |
| EA-SIc_7 | I receive appropriate ***material*** gifts (chocolates, bouquets of flowers, food, small lucky charms, etc.) from my colleagues. |
| EA-SIc_8 | The image my colleagues have of me matches what I do every day. |
| EA-SIc_9 | Sometimes I feel under pressure from my colleagues. |
| EA-SIc_10 | My colleagues make me feel that I am worth a lot. |
| EA-SIc_11 | My colleagues show personal and professional interest in me. |
| EA-SIc_12 | My colleagues enable me to learn new things. |
| EA-SIc_13 | My colleagues spare no expense or effort to create a working environment in which I can work well (working environment, materials, decoration and plants, etc.). |
| EA-SIc_14 | My colleagues also share personal and emotional topics with me. |
| EA-SIc_15 | My colleagues give me the feeling of being part of a community. |

Does not apply Applies I cannot answer

| | | | | | | | | | | |
|---|---|---|---|---|---|---|---|---|---|---|
| **O** | **O** | **O** | **O** | **O** | **O** | **O** | **O** | **O** | **O** | **O** |

*Note*. Please note that the original scale was developed in German. The scale has been translated into English for better understanding. The scale's statistical validation in English is pending.

## 8.3 Appendix C – Original EA-SI Work Scale – Supervisors

The following questions relate to the appreciation you experience from others in your day-to-day work. The important thing here is that it is about how much you personally feel valued. There are no right or wrong answers. Please answer honestly and conscientiously.

At this point, we are interested in your personal feelings and assessments. Below you will be presented with statements that relate to your direct manager. By "direct manager" we mean the manager with whom you work and have direct contact.

*Please indicate to what extent these statements apply to you personally. Remember specifically the last three months. Please tick the option "I cannot answer." only if it does not apply at all to your professional activity or cannot be answered.*

| | |
|---|---|
| EA-SIs_1 | My direct supervisor takes my objections seriously. |
| EA-SIs_2 | My direct supervisor shows me that she knows and appreciates my strengths. |
| EA-SIs_3 | My direct supervisor gives me responsibility for important company resources (financial resources, personnel, materials, etc.). |
| EA-SIs_4 | My direct supervisor always emphasises that she can rely on me. |
| EA-SIs_5 | I can clearly feel that my direct supervisor appreciates my work. |
| EA-SIs_6 | I feel that all my successes are recognised by my direct supervisor. |
| EA-SIs_7 | I receive appropriate material gifts (bonus payments, vouchers, chocolates, presents, etc.) from my direct supervisor. |
| EA-SIs_8 | The image that my direct supervisor has of me matches what I do every day. |
| EA-SIs_9 | Sometimes I feel under pressure from my direct supervisor. |
| EA-SIs_10 | My direct supervisor makes me feel that I am worth a lot. |
| EA-SIs_11 | My direct supervisor shows personal and professional interest in me. |
| EA-SIs_12 | My direct supervisor lets me know that she sees everything I do. |
| EA-SIs_13 | My direct supervisor spares no expense or effort to create a working environment in which I can work well (working environment, materials, technical equipment, required software, offers for joint company outings/activities, etc.). |
| EA-SIs_14 | I can talk to my direct supervisor about personal and emotional issues. |
| EA-SIs_15 | My direct supervisor gives me the feeling of being part of a community. |

Does not apply Applies I cannot answer

| | | | | | | | | | | |
|---|---|---|---|---|---|---|---|---|---|---|
| O | O | O | O | O | O | O | O | O | O | O |

*Note*. Please note that the original scale was developed in German. The scale has been translated into English for better understanding. The scale's statistical validation in English is pending.

## 8.4 Appendix D – Item Selection Prompt using ChatGPT

### *8.4.1 Original Prompt*

Wähle aus den folgenden Aussagen fünf Aussagen aus:

[VERBLIEBENE NEUN AUSSAGEN NACH ERSTER ITEMSELEKTION]

Diese sollen die folgende Kriterien erfüllen:

1. Möglichst vielseitige Abbildung von erfahrener Wertschätzung
2. Anerkennen der Leistungen und Arbeit der Person
3. Vermittlung eines hohen Wertes als Person
4. Verbundenheit in der Interaktion
5. Möglichst geringe inhaltliche Redundanz
6. Möglichst geringe sprachliche Redundanz

Bitte begründe deine Auswahl.

### *8.4.2 Translated Prompt*

Choose five statements from the following:

[REMAINING NINE ITEMS AFTER FIRST ITEM SELECTION]

These should fulfill the following criteria:

1. the most versatile representation of experienced appreciation possible
2. recognizing the person's achievements and work
3. conveying a high value as a person
4. connectedness in the interaction
5. as little content redundancy as possible
6. as little linguistic redundancy as possible

Please give reasons for your choice.

## 8.5 Appendix E – Misfit Plot for Colleagues – Original EA-SI Work Scale

### *8.5.1 Table E.1*

*Misfit Plots for Colleagues as a Source of EA-SI*

| | 1 | 2 | 3 | 4 | 5 | 6 | 7 | 8 | 9 | 10 | 11 | 12 | 13 | 14 | 15 |
|---|---|---|---|---|---|---|---|---|---|---|---|---|---|---|---|
| 1 | **0** | | | | | | | | | | | | | | |
| 2 | .07 | **0** | | | | | | | | | | | | | |
| 3 | .05 | .05 | **0** | | | | | | | | | | | | |
| 4 | .02 | .05 | .02 | **0** | | | | | | | | | | | |
| 5 | .01 | .04 | .02 | .03 | **0** | | | | | | | | | | |
| 6 | 0 | .03 | .01 | .02 | .01 | **0** | | | | | | | | | |
| 7 | .02 | .05 | 0 | .02 | .03 | .02 | **0** | | | | | | | | |
| 8 | .02 | .02 | .04 | 0 | .01 | .01 | .01 | **0** | | | | | | | |
| 9 | 0 | .02 | .07 | 0 | .04 | .01 | .02 | .06 | **0** | | | | | | |
| 10 | .02 | 0 | .02 | .01 | .01 | .02 | .01 | .02 | .01 | **0** | | | | | |
| 11 | .01 | .02 | .04 | .04 | .05 | .01 | 0 | .03 | .03 | .01 | **0** | | | | |
| 12 | .05 | .04 | .02 | .02 | .03 | .04 | .01 | .04 | .05 | 0 | .09 | **0** | | | |
| 13 | .02 | .03 | .04 | .08 | .02 | .02 | .11 | .04 | .01 | .02 | .02 | .05 | **0** | | |
| 14 | .01 | .01 | .04 | .04 | .04 | .02 | .04 | .03 | .06 | .03 | .03 | .07 | .01 | **0** | |
| 15 | 0 | 0 | .07 | 0 | .04 | .03 | .14 | .02 | .03 | .03 | .06 | 0 | 0 | .05 | **0** |

*Note* . The numbers represent the items in the order of the EA-SI Work Scale.

### *8.5.2 Table E.2*

*Misfit Plots for Direct Supervisors as a Source of EA-SI*

| | 1 | 2 | 3 | 4 | 5 | 6 | 7 | 8 | 9 | 10 | 11 | 12 | 13 | 14 | 15 |
|---|---|---|---|---|---|---|---|---|---|---|---|---|---|---|---|
| 1 | **0** | | | | | | | | | | | | | | |
| 2 | 0 | **0** | | | | | | | | | | | | | |
| 3 | .02 | 0 | **0** | | | | | | | | | | | | |
| 4 | .04 | .03 | .05 | **0** | | | | | | | | | | | |
| 5 | .03 | .01 | .03 | .01 | **0** | | | | | | | | | | |
| 6 | .03 | 0 | .02 | 0 | 0 | **0** | | | | | | | | | |
| 7 | .01 | .03 | .04 | .02 | .02 | .03 | **0** | | | | | | | | |
| 8 | .01 | .01 | .10 | .06 | .01 | .01 | .10 | **0** | | | | | | | |
| 9 | .03 | .01 | .10 | .01 | .03 | .03 | .03 | .06 | **0** | | | | | | |
| 10 | .02 | .01 | .01 | .02 | .02 | .01 | 0 | .03 | .01 | **0** | | | | | |
| 11 | .03 | .01 | .02 | .03 | .01 | .01 | .01 | 0 | .03 | .02 | **0** | | | | |
| 12 | .03 | .01 | 0 | .04 | .01 | .01 | 0 | .01 | .02 | .01 | .01 | **0** | | | |
| 13 | .04 | .04 | .06 | .03 | .01 | .03 | .14 | .11 | .01 | .01 | .02 | .04 | **0** | | |
| 14 | .02 | 0 | .05 | .03 | .04 | .04 | .03 | 0 | .06 | .01 | .10 | 0 | .02 | **0** | |
| 15 | .04 | .04 | .01 | 0 | .01 | .02 | .04 | .04 | .03 | .01 | .02 | .01 | .05 | .05 | **0** |

*Note* . The numbers represent the items in the order of the EA-SI Work Scale.

## 8.6 Appendix F – EA-SI Work Scale (short) – Colleagues

The following questions relate to the appreciation you experience from others in your day-to-day work. The important thing here is that it is about how much you personally feel valued. There are no right or wrong answers. Please answer honestly and conscientiously.

At this point, we are interested in your personal feelings and assessments. Below you will be presented with statements that relate to your colleagues.

*Please indicate to what extent these statements apply to you personally. Remember specifically the last three months. Please tick the option "I cannot answer." only if it does not apply at all to your professional activity or cannot be answered.*

| | |
|---|---|
| EA-SIc_2 | My colleagues show me that they know and appreciate my strengths. |
| EA-SIc_5 | I can clearly feel that my colleagues appreciate my work. |
| EA-SIc_10 | My colleagues make me feel that I am worth a lot. |
| EA-SIc_11 | My colleagues show personal and professional interest in me. |

Does not apply Applies I cannot answer

| | | | | | | | | | | |
|---|---|---|---|---|---|---|---|---|---|---|
| **O** | **O** | **O** | **O** | **O** | **O** | **O** | **O** | **O** | **O** | **O** |

*Note*. Please note that the original scale was developed in German. The scale has been translated into English for better understanding. The scale's statistical validation in English is pending.

## 8.7 Appendix G – EA-SI Work Scale (short) – Supervisors

The following questions relate to the appreciation you experience from others in your day-to-day work. The important thing here is that it is about how much you personally feel valued. There are no right or wrong answers. Please answer honestly and conscientiously.

At this point, we are interested in your personal feelings and assessments. Below you will be presented with statements that relate to your direct manager. By "direct manager" we mean the manager with whom you work and have direct contact.

*Please indicate to what extent these statements apply to you personally. Remember specifically the last three months. Please tick the option "I cannot answer." only if it does not apply at all to your professional activity or cannot be answered.*

| | |
|---|---|
| EA-SIs_2 | My direct supervisor shows me that she knows and appreciates my strengths. |
| EA-SIs_5 | I can clearly feel that my direct supervisor appreciates my work. |
| EA-SIs_10 | My direct supervisor makes me feel that I am worth a lot. |
| EA-SIs_11 | My direct supervisor shows personal and professional interest in me. |

Does not apply Applies I cannot answer

| | | | | | | | | | | |
|---|---|---|---|---|---|---|---|---|---|---|
| **O** | **O** | **O** | **O** | **O** | **O** | **O** | **O** | **O** | **O** | **O** |

*Note*. Please note that the original scale was developed in German. The scale has been translated into English for better understanding. The scale's statistical validation in English is pending.

## 8.8 Appendix H – Misfit Plots – EA-SI Work Scale (short)

### *8.8.1 Table H.1*

*Misfit Plots for Colleagues as a Source of EA-SI*

| | Item 10 | Item 5 | Item 2 | Item 11 |
|---|---|---|---|---|
| Item 10 | 0 | | | |
| Item 5 | .01 | 0 | | |
| Item 2 | .02 | 0 | 0 | |
| Item 11 | .01 | .02 | .03 | 0 |

*Note*. The numbers represent the items in the order of the EA-SI Work Scale (short).

### *8.8.2 Table H.2*

*Misfit Plots for Supervisors as a Source of EA-SI*

| | Item 10 | Item 5 | Item 2 | Item 11 |
|---|---|---|---|---|
| Item 10 | 0 | | | |
| Item 5 | 0 | 0 | | |
| Item 2 | .01 | 0 | 0 | |
| Item 11 | 0 | .02 | .04 | 0 |

*Note*. The numbers represent the items in the order of the EA-SI Work Scale (short).

## 8.9 Appendix I – Descriptive Analyses in Study Two

| | M | SD | Range | Internal Consistency | EA-SI Colleagues | EA-SI Supervisors | Self-Esteem | Perceived Stress | Appreciation Colleagues single | Appreciation Supervisors single | Environmental Politics | Work Engagement | Burnout |
|---|---|---|---|---|---|---|---|---|---|---|---|---|---|
| 1 | 7.63 | 1.63 | 7.86 | .89 | — | | | | | | | | |
| 2 | 6.87 | 2.39 | 9.00 | .95 | 0.48** | — | | | | | | | |
| 3 | 3.81 | 0.87 | 4.00 | .88 | 0.37** | 0.25** | — | | | | | | |
| 4 | 4.72 | 1.76 | 6.00 | .86 | –0.35** | –0.26** | –0.48** | — | | | | | |
| 5 | 2.53 | 0.86 | 3.75 | — | 0.75** | 0.38** | 0.22** | –0.30** | — | | | | |
| 6 | 4.64 | 0.82 | 3.90 | — | 0.40** | 0.89** | 0.20** | –0.27** | 0.39** | — | | | |
| 7 | 2.29 | 0.61 | 2.87 | .90 | 0.08 | –0.05 | 0.02 | –0.04 | 0.04 | –0.06 | — | | |
| 8 | 4.57 | 1.32 | 5.89 | .94 | 0.41** | 0.37** | 0.26** | –0.33** | 0.34** | 0.34** | 0.03 | — | |
| 9 | 2.93 | 1.28 | 5.78 | .90 | –0.39** | –0.43** | –0.39** | 0.55** | –0.36** | –0.46** | 0.06 | –0.47** | — |

*Note.* Mean (*M*), Standard Deviation (*SD*), Range, and Cronbach's Alpha ($\alpha$) have been computed. $p < .05$ = *, and $p < .01$ = **. All results refer to EA-SI measured with the newly developed schort scale.

## 8.10 Appendix J – Data Transparency Appendix – Study One

| **Variables in the Complete Dataset** | **Poster (STATUS = presented)** | **MS 1 (STATUS = under review)** | **MS 2 (STATUS = current)** | **MS 3 (STATUS = planned)** |
|---|---|---|---|---|
| **Experienced Appreciation (Colleagues)** | | **X** | **X** | **X** |
| **Experienced Appreciation (Supervisors)** | **X** | **X** | **X** | **X** |
| **Perceived Stress** | **X** | **X** | | **X** |
| **Global Self-Esteem** | **X** | **X** | | **X** |
| **Work Satisfaction** | **X** | **X** | | **X** |
| **Life Satisfaction** | **X** | **X** | | |
| **Work Engagement** | **X** | **X** | | **X** |
| **Emotional Exhaustion** | **X** | **X** | | |
| **Appreciation at Work Scale** | | **X** | | |
| **Appreciation single Item (Colleagues)** | | **X** | | |
| **Appreciation single Item (Supervisors)** | | **X** | | |
| **Workplace Ostracism** | | **X** | | |
| **Interpersonal Justice** | | **X** | | |
| **Political Environmental Decisions** | | **X** | | |
| **Social Support** | | **X** | | |
| **Big Five Inventory** | | | | **X** |
| **Turnover Intention** | | | | **X** |
| **Sleep Quality** | | | | |
| **Effort-Reward Imbalance** | | | | |

*Note.* The abbreviation MS stands for manuscripts planned or written using Study One’s data.

## 8.11 Appendix K – Data Transparency Appendix – Study Two

| **Variables in the Complete Dataset** | **MS 2 (STATUS = current)** | **No further MS planned** |
|---|---|---|
| **Experienced Appreciation (Colleagues)** | **X** | |
| **Experienced Appreciation (Supervisors)** | **X** | |
| **Perceived Stress** | **X** | |
| **Global Self-Esteem** | **X** | |
| **Work Engagement** | **X** | |
| **Burnout** | **X** | |
| **Appreciation single Item (Colleagues)** | **X** | |
| **Appreciation single Item (Supervisors)** | **X** | |
| **Political Environmental Decisions** | **X** | |
| **Social Support** | **X** | |
| **Turnover Intention** | | |

*Note.* The abbreviation MS stands for manuscripts planned or written using Study Two's data.
